# Anopheline species composition and the 1014F-genotype in different ecological settings of Burkina Faso in relation to malaria transmission

**DOI:** 10.1186/s12936-019-2789-8

**Published:** 2019-05-08

**Authors:** Alphonse Traoré, Athanase Badolo, Moussa W. Guelbeogo, Antoine Sanou, Mafalda Viana, Luca Nelli, Soumanaba Zongo, Hyacinthe K. Toé, Alfred S. Traoré, Hilary Ranson, N’Falé Sagnon

**Affiliations:** 1 Centre National de Recherche et de Formation sur le Paludisme, 01 BP 2208 Ouagadougou 01, Burkina Faso; 2 Laboratoire d’Entomologie Fondamentale et Appliquée, Université Ouaga1 Pr Joseph Ki-Zerbo, BP 7021 Ouagadougou 03, Burkina Faso; 3 Department of Vector Biology, Liverpool School of Tropical Medicine, Pembroke Place, Liverpool, L3 5QA UK; 4 Institute of Biodiversity, Animal Health and Comparative Medicine, College of Medical, Veterinary and Life Sciences, University of Glasgow, University Avenue, Glasgow, G12 8QQ UK

**Keywords:** Malaria, Diversity, *Anopheles gambiae*, *Plasmodium falciparum*, *kdr*, Insecticide, Resistance

## Abstract

**Background:**

A three-year longitudinal study was conducted in four sentinel sites from different ecological settings in Burkina Faso, between 2008 and 2010 to identify longitudinal changes in insecticide resistance within *Anopheles gambiae* complex species based on larval collection. During this study, adult mosquitoes were also collected indoor and outdoor using several methods of collection. The present study reports the diversity of malaria vectors and the 1014F-genotype from this adult collection and investigates the association between this 1014F-genotype and sporozoite rate.

**Methods:**

Adult mosquitoes were collected from July to August (corresponding to the start of rainy season) and October to November (corresponding to the end of rainy season) over 3 years (2008–2010) at four sites across the country, using pyrethrum spray catches (PSC), exit traps and pit shelters. *Anopheles gambiae* complex mosquitoes were identified to species and genotyped for the L1014F *kdr* mutation by PCR using genomic DNA. The circumsporozoite antigen of *Plasmodium falciparum* was detected in mosquitoes using sandwich ELISA.

**Results:**

Overall 9212 anopheline mosquitoes were collected during the study period. Of those, 6767 mosquitoes were identified as *Anopheles gambiae* sensu lato (s.l.). *Anopheles arabiensis, Anopheles coluzzii, Anopheles gambiae* and or *Anopheles funestus* were incriminated as vectors of *P. falciparum* in the study area with an average sporozoite rate of 5%, (95% CI 4.14–5.99%). The *kdr*1014F-genotype frequencies were 11.44% (95% CI 2.5–39.85%), 19.2% (95% CI 4.53–53.73%) and 89.9 (95% CI 63.14–97.45%), respectively for *An. arabiensis, An. coluzzii* and *An. gambiae.* The proportion of the 1014F-genotype varied between sporozoite-infected and uninfected *An. gambiae* s.l. group. There was no significant difference in the 1014F-genotype frequency between infected and uninfected mosquitoes.

**Conclusion:**

The current study shows the diversity of malaria vectors and significant interaction between species composition and *kdr*1014F-genotype in *An. gambiae* complex mosquitoes from Burkina Faso. In this study, no associations were found between the 1014F-genotype and *P*. *falciparum* infection in the major malaria vector *An. gambiae* s.l.

**Electronic supplementary material:**

The online version of this article (10.1186/s12936-019-2789-8) contains supplementary material, which is available to authorized users.

## Background

After decades of efforts to malaria control, this disease is still a major public health concern in sub-Saharan Africa, responsible for an estimated of 219 million cases and 435,000 deaths in 2017 [1]. Burkina Faso is a malaria endemic country with 19 million inhabitants, where nearly 12 million malaria cases were reported in 2017 with 4144 resulting in death [2]. In Burkina Faso, malaria prevention primarily relies on insecticide-treated bed nets. In 2010, 2013 and 2016 combined, approximately 36 million long-lasting insecticide-treated nets (LLINs) were freely distributed through mass distribution campaigns. Additional sporadic measures such as Indoor Residual Spraying (IRS) with bendiocarb were introduced in 2010 in Diébougou, a locality situated in the southwestern part of Burkina Faso but this pilot programme was terminated in 2011 (http://www.africairs.net/where-we-work/burkina-faso/). A larviciding pilot programme using *Bacillus thuringiensis* was also implemented for 1 year in 2012 to control malaria in Ouagadougou the capital city of Burkina Faso.

*Anopheles gambiae*, *Anopheles coluzzii*, *Anopheles arabiensis* and *Anopheles funestus* are the most important malaria vectors in Burkina Faso [3, 4]. Members of the *An. gambiae* complex are sympatric species with different ecological niches [5, 6]. Previous studies showed that the species composition of the *An. gambiae* complex varied across Burkina Faso climatic conditions. Whilst *Anopheles arabiensis* and *An. coluzzii* are distributed with equal frequency in central and eastern regions of the country [7], the western regions, where rainfall is abundant, are dominated by *An. gambiae* sensu stricto (s.s) [5, 8]. *Anopheles arabiensis* is the most abundant species found in urban compared to rural areas [6]. Recently a new cryptic sub-group inside *An. gambiae*, named Goundry, was identified in Burkina Faso [9] with an exophilic behaviour.

The development of resistance to insecticides in malaria vectors is one of the main concerns for malaria control as to date major vector tools rely on insecticide use. In Burkina Faso, resistance to the four major classes of insecticides (organochlorides, organophosphates, pyrethroids and carbamates) used in public health has recently increased throughout the country. The resistance level to pyrethroids is particularly high in the Western region of the country [10–12], which may affect bed nets efficacy [13]. Four mechanisms of insecticide resistance have been described in West Africa [14, 15]. One of the most widespread mechanisms is target site mutation associated with resistance to pyrethroids and DDT [16] in the voltage-gated sodium channel, associated with knock down resistance (*kdr)* alleles. Three *kdr* mutations have been reported in the *An. gambiae* complex: L1014F, L1014S and N1575Y [17–19]. There is increasing evidence that insecticide pressure used in public health and agriculture is leading to the selection of insecticide resistance in malaria vectors [13]. The 1014F *kdr* allele frequency is very high in *An. gambiae* and *An. coluzzii* in Burkina Faso, while the 1014S is very common in *An. arabiensis* [10, 12, 20].

There is little empirical data on the impact of insecticide resistance on malaria transmission but modeling has predicted that widespread resistance to pyrethroids will result in additional 260,000 deaths in children under 5 years of age [21] in the WHO African Regions. Insecticide resistance is assumed to increase the likelihood of mosquito-borne disease transmission by increasing the vector population size and allowing mosquitoes a long period of life even the presence of insecticides [22]. On the other hand, resistance may reduce the vectorial capacity by imposing a major fitness cost to mosquitoes [22], but laboratory experiments have suggested that mosquito strains with the *kdr* mutation were more susceptible to *Plasmodium* infection [23]. However, it has also been reported that older *Anopheles* mosquitoes are more susceptible to insecticides than newly-emerged ones [24–27]. Hence, if insecticide resistant mosquitoes are more susceptible to infection with the malaria parasite, then the chances to transmit malaria parasite in areas with intensive insecticide exposure may be diminished, according to Viana et al. [28]. In contrast, a previous laboratory experiments showed that mosquitoes resistant to pyrethroids may be more susceptible to *P. falciparum* infection, and thus could potentially be more efficient malaria vectors [29]. Thus, field studies are needed to establish whether there is any association between insecticide resistance and the ability of the mosquito to transmit the malaria parasite (i.e. the presence of sporozoites in the mosquito’s salivary glands).

A three-year longitudinal study was conducted in Burkina Faso between 2008 and 2010 to assess for longitudinal changes in insecticide resistance from larval collections and results were published in Badolo et al. [10]. In addition, during this study, adult mosquitoes were also collected indoor and outdoor in different ecological settings with different levels of insecticide resistance. The objectives of this work were to report on the species diversity of malaria vectors in these different ecological settings of Burkina Faso and the association between insecticide resistance and the mosquito’s ability to transmit malaria. Due to the sample size it was not possible to investigate the association between L1014F-mutation and sporozoite infection. Instead, this study assessed the association between L1014F-genotype with the sporozoite infection.

## Methods

### Study area and mosquito collections

In 2008, four sentinel sites were established in Burkina Faso according to their different patterns of insecticide use and ecology. These sites include Goundry (rural Sahelian area with a low insecticide use), Koupela (Sahelian rural zone with moderate insecticide used), Soumousso (Sudan Savannah rural zone with rice and cotton cultivation using intensive insecticides) and Kuinima (urban area with intensive use of insecticide in agriculture). From these sites mosquito samples were collected, as previously described by Ranson et al. [11]. In each locality, a window exit trap was installed in five houses, with the homeowners’ permission. Volunteers were trained to empty the trap daily into tubes containing silicagel. At the same time two pit-shelters were built in the site, consisting of a deep well with a straw roof, for collecting the outdoor-resting mosquitoes. Indoor pyrethroid spray catches (PSC) were also performed in ten randomly selected houses from each site once per month to sample indoor-resting mosquitoes. Mosquitoes were collected at the beginning of the rainy season (July–August) corresponding to the beginning of malaria transmission and at the end of rainy season (September–October), which corresponds to the high malaria transmission period.

### Species identification

Mosquitoes were sorted morphologically according to the identification keys described by Edwards [30] for Culicine, and by Gillies and Coetzee [31] for Anopheline. All mosquitoes belonging to the *An. gambiae* complex were stored in silica gel to be used later for molecular analysis.

### Molecular analysis for *Anopheles gambiae* complex members’ identification and *Plasmodium falciparum* antigen protein detection

The head-thoraces from 2178 females *An. gambiae* sensu lato (s.l.) were used to assess for the presence of *P. falciparum* circumsporozoite protein (CSP) antigen according to method described by Wirtz et al. [32]. In addition to the ELISA-CSP detection, genomic DNA was extracted from the same individual mosquito samples and tested using a modified assay described by Paskewitz and Collins [33], then used for species identification, based on PCR–RFLP (restriction fragment length polymorphism) method described by Fanello et al. [34].

### Molecular detection of the L1014F mutation

All ELISA positive samples (182 mosquitoes) and an equal number of negative samples randomly chosen within the same collection was genotyped for the L1014F *kdr* mutation. The primers Agd1 (5′-ATAGATTCCCCGACCATG-3′) and Agd3 (5′-AATTTGCATTACTTACGACA-3′) were used for the detection of the mutant 1014F allele whereas primers Agd2 (5′-AGACAAGGATGATGAACC-3′) and Agd4 (5′-CTGTAGTGATAGGAAATTTA-3′) for the detection of the wild-type, 1014L allele [17]. The PCR program was 95 °C/5 min × 1 cycle, (95 °C/30 s, 46 °C/30 s, 72 °C/15 s) × 35 cycles, 72 °C/5 min × 1 cycle, and maintain at 4 °C after the PCR is completed.

### Data analysis

All data analysis was performed using the Generalized Linear Mixed Effect Models (GLMMs) through the R statistical software version 3.5.0 (2018-04-23) [35]. Model selection was based on Likelihood Ratio Tests (LRTs) using back-elimination, i.e. starting with a full model. The mean predicted values and 95% confident intervals for significant terms were computed using the “effect” package [36].

### Mosquito species abundance

As the abundance data were over-dispersed, the drivers of mosquito abundance were investigated through a negative binomial distribution. Here the mosquito counts were included as the response variable and site, collection method and year were used as explanatory factors. Because these factors are expected to differ among species, an interaction term of species with all three factors was included. Collection date and compound from which collection took place were added to the model as random effects.

### Sporozoite rate calculation

The proportion of infected mosquitoes with *P. falciparum* sporozoites was modeled following a binomial distribution. This proportion was used as response variable whereas the species, site and method were included as explanatory variables. Collection date was used as a random effect.

### Association between sporozoite rate and 1014F-genotypes

To assess for the differences in resistance between infected and uninfected mosquitoes, a binomial distribution was used. Individuals mosquitoes were considered as resistance genotype (homozygote L1014F) or susceptible genotype (including heterozygote L1014F and homozygote L1014L) since that the *kdr* gene is recessive [37]. As the sporozoite rate was controlled by deliberately selecting an equal number that were infected and uninfected, genotype (RR) was used as a binary response variable and species while CSP were included as explanatory variable when controlling for method, year and site used as random effects variables. Due to the low number of replicates, year, sites and trapping method were included as random effects.

## Results

### Mosquito species abundance and composition

**Fig. 1:**
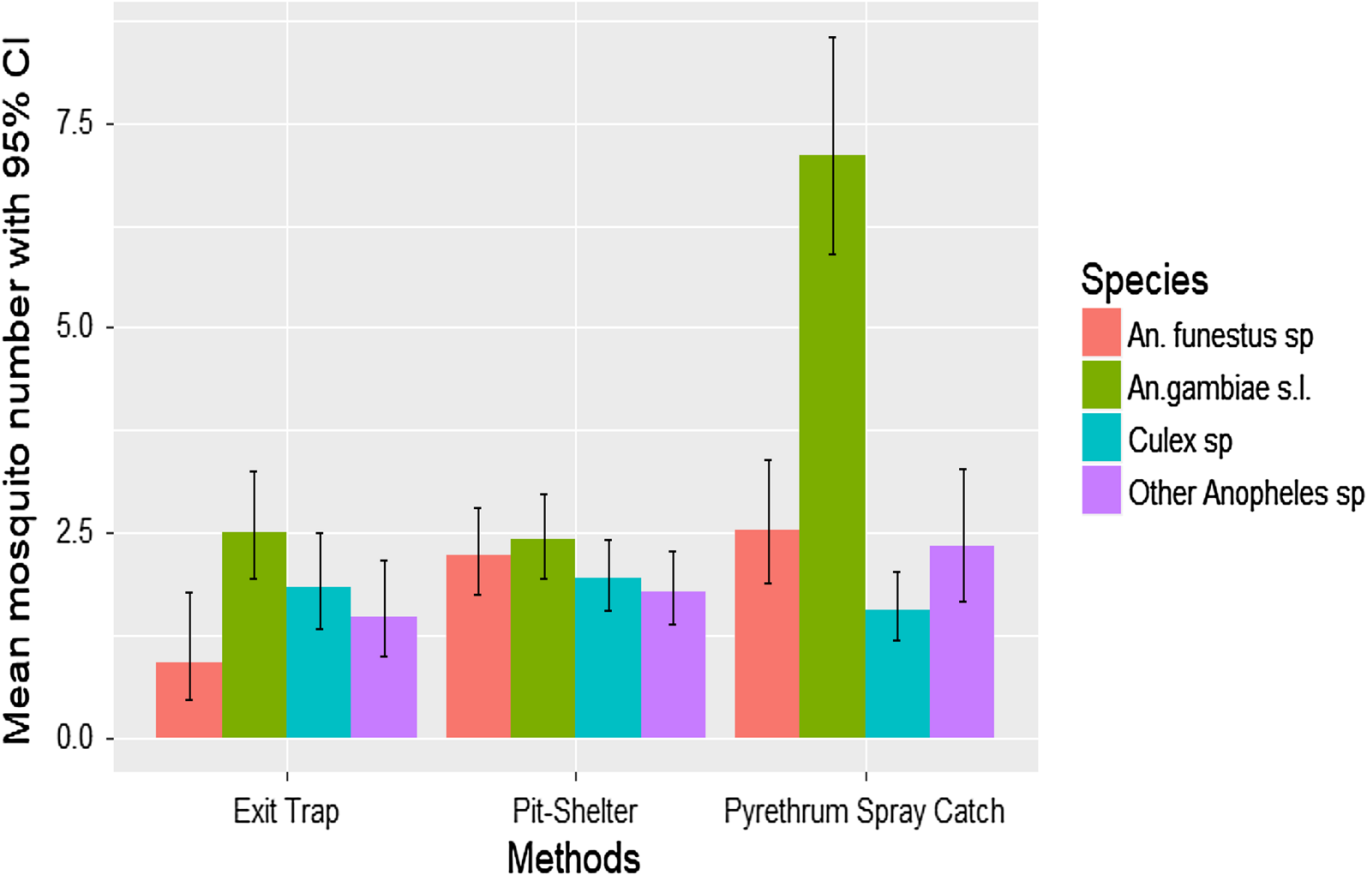
Mean number of mosquitoes predicted by the model by methods and per species over sites and years

**Fig. 2:**
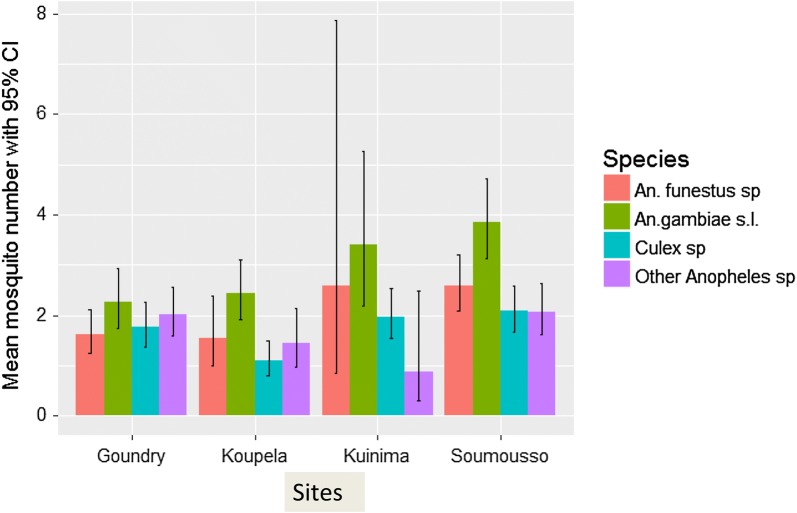
Mean number of mosquitoes predicted by the model by sites and per species over tapping methods and years

From July 2008 to November 2010 10,938 mosquitoes were collected in the four sites. Of those, 9212 (84.22%) were Anopheline, followed by *Culex sp.* 1678 (15.34%), *Aedes sp.* 31 (0.33%) and *Mansonia sp.*11 (0.1%). From the 9212 Anopheline collected, 6767 (73.46%) were *An. gambiae* s.l., 1554 (16.87%) were *An. funestus* and the remaining 891 (8.15%) consisted of other Anopheline, including *Anopheles brucei* (0.22%), *Anopheles nili* (0.79%), *Anopheles coustani* (0.56%), *Anopheles pharoensis* (0.22%) and *Anopheles rufipes* (90.01%). For the data analysis these latest species were grouped under ‘other species’ because of their low number. For the abundance data analysis, the final model included the three interactions between species and year, method and sites. The mean number of mosquitoes collected by each method in the study areas is shown in (Figs. 1, 2). The difference between methods of collection was statistically significant (χ^2^ = 135.13, df = 6, P < 0.001). *Anopheles gambiae* s.l. was the most abundant species observed in PSC and exit tap collection methods compared to *An. funestus* and other Anopheline (Additional file 1: Table S1). The ratio between *An. gambiae* s.l. and *An*. *funestus* was 0.379 in PSC (P < 0.001) and 0.391 in exit traps (P = 0.04) collections, respectively. In contrast, there was no significant difference between *An. gambiae* s.l. and *An. funestus* collected from the pit shelters with ratios of 0.979(P > 0.05).

### *Anopheles gambiae* complex species diversity according to collection methods, years and sites

**Fig. 3:**
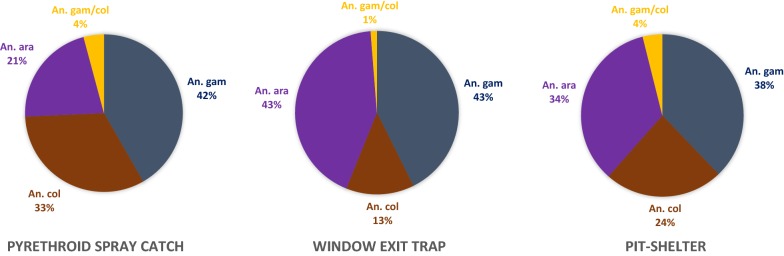
Distribution of *Anopheles gambiae* complex species by collection methods. The proportion of each species is provided in percentage. *An. ara, Anopheles arabiensis*; *An. col, Anopheles coluzzii*; *An. gam *=* Anopheles gambiae; An*. *gam*/*col *= hybrid *Anopheles gambiae*/*Anopheles coluzzii*

Molecular diagnostic assays showed that the *An. gambiae* complex includes *An. arabiensis*, *An. coluzzii* and *An. gambiae*. The relative frequencies were 29.95% for *An. arabiensis* (n = 660), 25.78% for *An. coluzzii* (n = 568), and 40.80% for *An. gambiae* (n = 899), with a few number of hybrid *An. gambiae/An. coluzzii* 3.45% (n = 76). *Anopheles gambiae* was the most predominant (χ^2^ = 116.61, df = 6, P < 0.001) from each trapping method collection (Fig. 3).

**Fig. 4:**
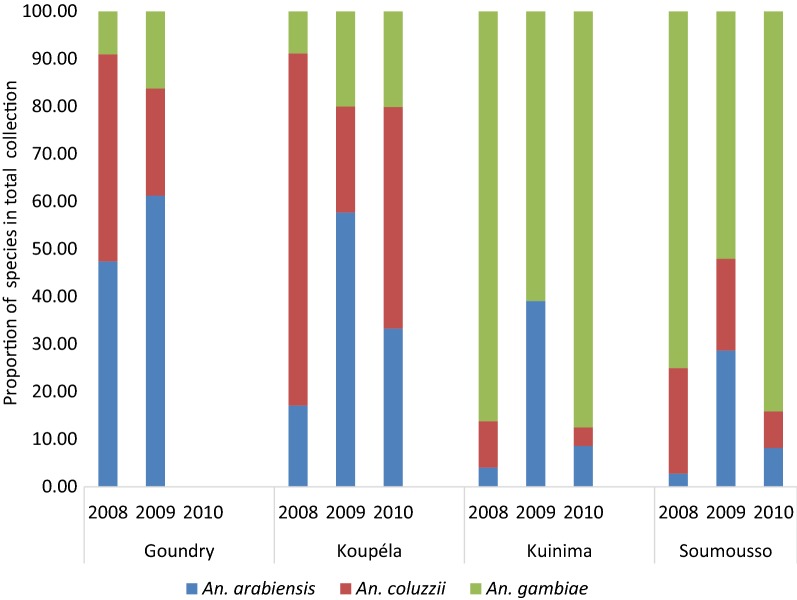
Distribution of *Anopheles gambiae* complex species by year and per site. The proportion of each species is provided in percentage

Overall, there was a significant difference in species composition between the three collection methods when pooling data across all four sites, (χ^2^ = 115, P < 0.001 (Additional file 2: Table S2). In Goundry, *An. arabiensis* was the predominant species (44.54%) with the higher proportion being collected by PSC followed by exit traps. In Koupela, the two predominant species were *An. coluzzii* and *An. arabiensis,* which were collected with similar proportions. However, in this area, *An. arabiensis* was found in higher proportion in exit traps followed by pit shelters and PSC, while *An. coluzzii* was more abundant in collections with PSC, followed by pit shelters and exit traps. In contrast, in Kuinima and Soumousso the predominant species was *An. gambiae* in all years of the study. Overall, *An. arabiensis* and *An. coluzzii* fluctuated in the sudan sahelian (Koupela) and sahelian (Goundry) zones (Fig. 4).

### *Plasmodium falciparum* sporozoite rate in *Anopheles gambiae* populations

**Fig. 5:**
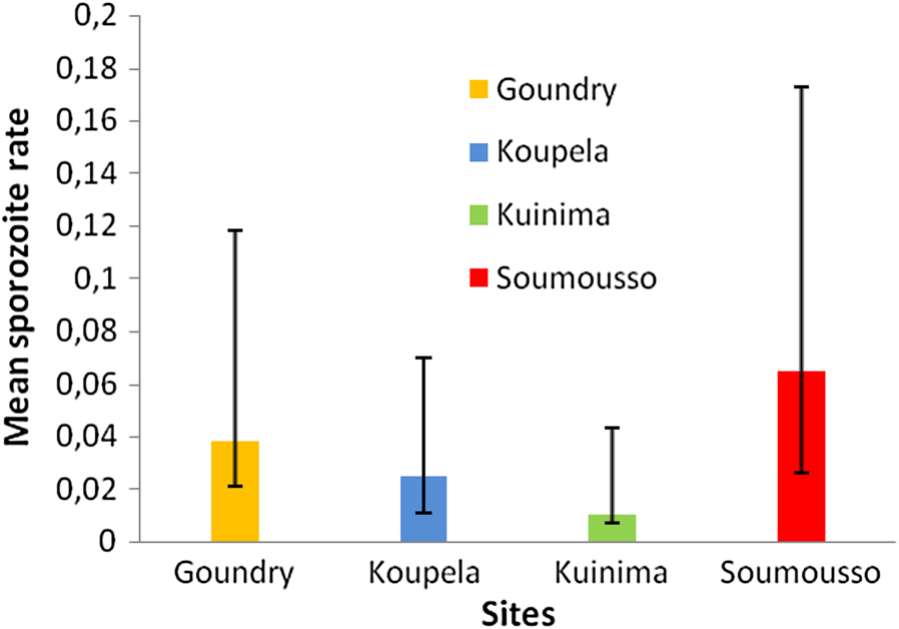
Mean sporozoite rate predicted in the study areas (Goundry, Koupela, Kuinima and Soumousso)

The best model included site, methods and species as explanatory variables. 110 mosquitoes out of 2178 were tested positive for *P*. *falciparum* CSP antigen resulting in a mean sporozoite rate of 0.050, (95% CI 0.041–0.060) (Table 1). The *P. falciparum* sporozoite rate varied significantly between the four sites of study area (Fig. 5; χ^2^ = 16.642 df = 3, P = 0.001), with the higher sporozoite rate of 0.065, (95% CI 0.039–0.108) observed in the site of Soumousso and the lower in Kuinima 0.010, (95% CI 0.003–0.031). Between species the sporozoite rate was higher for *An. gambiae* 0.065, (95% CI 0.049–0.083) compared to *An. coluzzii* 0.044, (95% CI 0.029–0.064) and *An. arabiensis* 0.038, (95% CI 0.025–0.055). However, there was no statistically significant difference of sporozoite rate between species within the *An. gambiae* complex (χ^2^ = 5.481, df = 2, P = 0.065).

**Table 1:** Detection of CSP antigen in Anophelines collected in the four sites of study

Locality	*An. arabiensis*		*An. coluzzii*		*An. gambiae*		*An. funestus*		Total	
	Tested	CSP+	Tested	CSP+	Tested	CSP+	Tested	CSP+	Tested	CSP+
Goundry	161	7	100	2	37	3	0	0	298	12
Koupéla	334	5	333	10	140	13	0	0	807	28
Kuinima	34	1	17	0	246	1	4	0	301	2
Soumousso	128	12	120	13	477	42	47	1	772	68
Spz rate	3.81%		4.39%		6.56%		1.96%		5.0%	
Total	657	25	570	25	900	59	51	1	2178	110

The difference in infection rate between trapping methods was not statistically significant (χ^2^ = 4.221, df = 2, P = 0.121) but the sporozoite rate was highest in PSC collections (mean: 0.060, (95% CI 0.047–0.076) and lowest in pit shelter collections (mean: 0.028, (95% CI 0.017–0.045). The *P*. *falciparum* sporozoite rate in exit traps was 0.056(95% CI 0.037–0.080).

### Association between sporozoite rate and 1014F-genotype

A total of 164 *An. gambiae* s.l. species were successfully genotyped for *kdr*1014F allele, from which 92 were resistant (RR) and 72 susceptible (SS and RS) (Table 2). The best model includes only species as explanatory fixed effect of the *kdr* resistant genotype (χ^2^ = 64.242, df = 2, P < 0.001). Therefore, the sporozoite infection status was not retained as a significant term (χ^2^ = 0.031, df = 1, P = 0.86).

**Table 2:** kdr genotype in *An. gambiae s.l* species collected in the four sites

Species	Resistant	Susceptible	Total
*An. arabiensis*	9	39	48
*An. coluzzii*	13	27	40
*An. gambiae*	70	6	76
Total	92	72	164

**Fig. 6:**
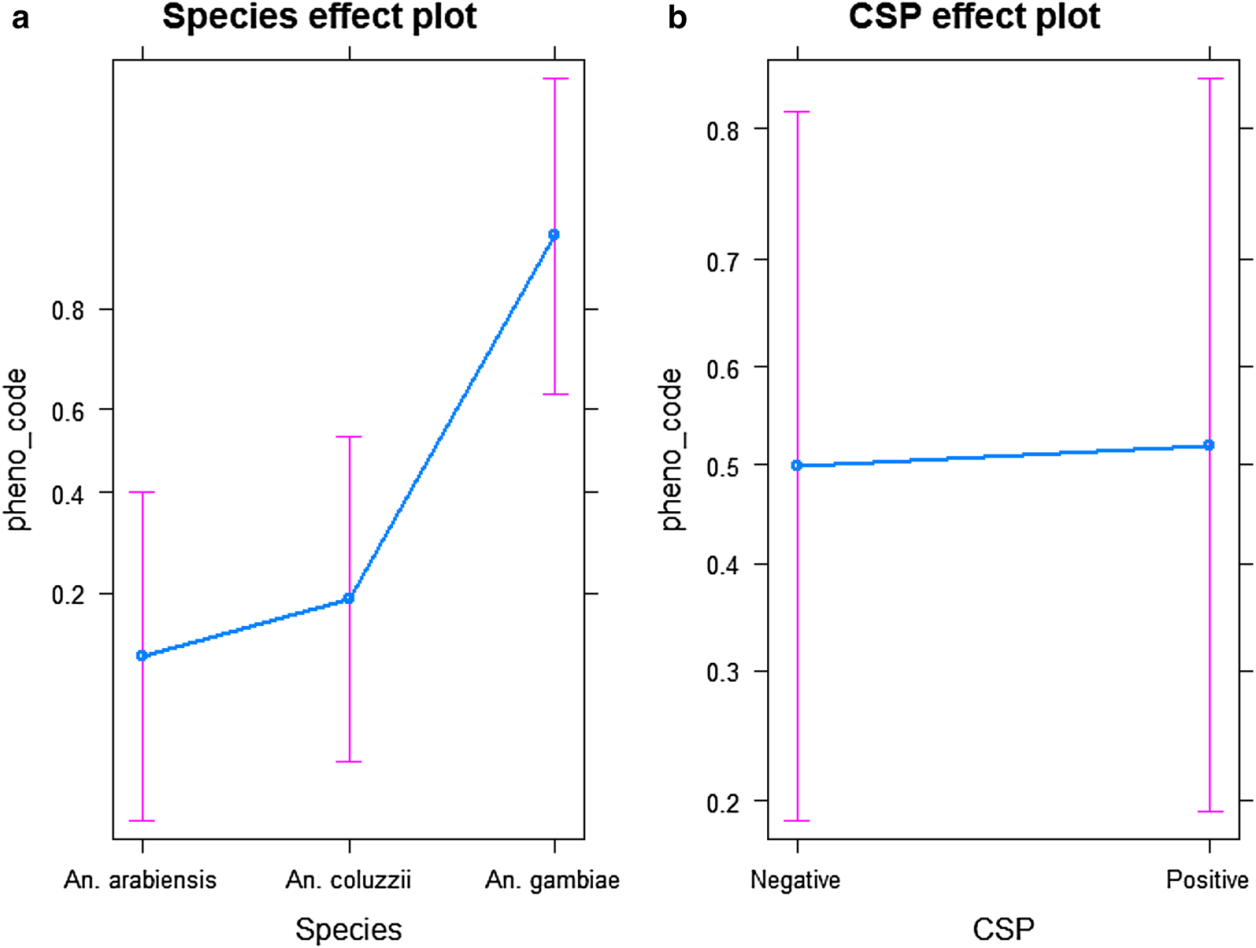
The effects of *An.* *gambiae* s.l. species composition (**a**) and the proportion of *kdr* resistant genotype in CSP-positive and CSP-negative mosquito’s (**b**)

There was no significant difference in the proportion of RR between *An. arabiensis* and *An. coluzzii* (OR = 0.575, P = 0.555). In contrast there was a significant difference in this proportion when comparing *An. arabiensis* versus *An. gambiae* (OR = 0.016, P < 0.001) and *An. coluzzii* versus *An. gambiae* (OR = 0.028, P < 0.001), (Fig. 6a, Additional file 3: Table S3).

In addition, the proportion of *kdr* resistant genotype did not varied when comparing sporozoite infected vs uninfected within each species, (χ^2^ = 0.035, P = 0.85) for *An. arabiensis*, (χ^2^ = 0.020, P = 0.88) for *An. coluzzii* and (χ^2^ = 0.006, P = 0.93) for *An. gambiae* (details are shown in Table 3).

**Table 3:** Relative proportions of *kdr 1014F*-genotype with 95% CI in CSP + and CSP- mosquitoes for *An. arabiensis*, *An. coluzzii* and *An. gambiae*

Status	Species	*An. arabiensis*		*An. coluzzii*		*An. gambiae*	
	n	Resistant	Susceptible	Resistant	Susceptible	Resistant	Susceptible
CSP-negative	98	6.12% (2.27–12.85)	26.54% (18.12–36.4)	8.16% (3.59–15.45)	20.41% (12.93–29.74)	35.71% (26.28–46.02)	3.06% (0.63–8.68)
CSP-positive	66	4.54% (0.94–12.71)	19.69% (10.92–31.32)	7.57% (2.5–16.8)	10.6% (4.37–20.63)	53.03% (40.34–65.43)	4.54% (0.94–12.71)
*Kdr* frequency		11.44% (2.5–39.85)		19.20% (4.53–53.73)		89.90% (63.14–97.45)	

Furthermore, within the sporozoite-infected sub-group the predicted proportion of resistant genotype was 0.52, with (95% CI 0.19–0.83) whilst 0.49, with (95% CI 0.19–0.81) within the uninfected (Fig. 6b).

## Discussion

The present study shows that *An. gambiae* s.l. is the most predominant malaria vector within the study area. Molecular identification showed that *An. gambiae* s.l. consists of the three species *An. gambiae* sensu stricto*, An. coluzzii* and *An arabiensis* and that there is a significant difference in their proportion between the sites within the study area. *Anopheles arabiensis* was the most abundant species occurring in Goundry, but found at the similar frequency as *An. Coluzzii* in Koupela. In the southern areas (Kuinima and Soumousso) *An. gambiae* s.s was the most predominant species followed by *An. arabiensis*. These findings are in agreement with previous studies suggesting that *An. gambiae* s.s*., An coluzzii* and *An. arabiensis* are the most widespread malaria vectors across Burkina Faso [6, 7]. However, the relative frequency of these species varied according to ecological settings. Furthermore, a significant reduction was found in the *An. arabiensis* proportion in 2008 from sample collected as adults within all sites particularly in Koupela, Kuinima and Soumousso compare to proportion from sample collected as larvae and reared to adulthood in laboratory conditions and described in Badolo et al. [10]. The missing proportion of *An. arabiensis* was subsequently filled mainly by *An. gambiae* s.s. in Soumousso and Kuinima and by *An. coluzzii* in Koupela and Goundry. The observed variation in species composition between larvae and adult populations may be partially due to the high susceptibility of *An. arabiensis* to insecticides as also suggested by the bioassay results described elsewhere showing an association between the 1014F-genotype and mosquito survival to permethrin and DDT [10]. This may be indicative of the effect of insecticide resistance on species composition according to insecticide use within a given area.

Complementary trapping methods were used to collect a representative sample of *Anopheles gambiae* complex mosquitoes with reference to resting behaviour. The results show a significant difference in species composition between these collection methods. In most cases, the three species from the *An. gambiae* complex were abundantly caught inside houses. An important fraction of this population was also found in exit traps and pit shelters. More specifically, *An. arabiensis* predominated in outdoor collection (window exit trap and pit shelters). This behaviour of *An. arabiensis* is consistent with previous studies showing that *An. arabiensis* are more generalist in terms of host choice and resting behaviour due to phenotypic plasticity [38].

Vector species with a relatively broad host range, like *An. arabiensis*, are thought to be better able to persist in areas of high indoor insecticide use. In contrast, *An. coluzzii* was caught in higher abundances in PSC. The PSC is intended to collect mosquitoes that feed and rest indoors or feed outdoors and indoors (endophilic mosquitoes), therefore, it may be less sensitive in collecting species that predominantly rest outdoors [39], compared to exit traps and pit shelters that target outdoor-resting mosquitoes. *Anopheles funestus* was highly caught indoor using PSC and outdoor in pit shelters. This finding is in agreement with previous studies on *An. funestus* population from Burkina Faso describing two chromosomal forms with different resting behaviour patterns: one form was mainly exophilic and the second endophilic [40].The current study shows significant difference in resting behaviour between *An. arabiensis* and the two other species, *An. coluzzii* and *An. gambiae* s.s., depending on the geographical location. Meanwhile the efficacy of vector control intervention based on IRS may be affected by this behaviour. This result is aligned with previous findings showing that *An. arabiensis* was more exophilic, exophagic and zoophilic than *An. gambiae* s.s [41].

Following Badolo et al. [10], one of the aims of this study was also to investigate the association between the *kdr* 1014F-genotype and the infection status. The results from this study showed that *P*. *falciparum* sporozoite rate varied significantly between the four study sites [10]. Based on laboratory experiments, Ndiath et al. [42] suggested that *An. coluzzii* was less susceptible to *P. falciparum* infection than *An. gambiae* s.s, while Gneme et al. [43] found that in Burkina Faso *An. gambiae* s.s. and *An*. *coluzzii* were equally susceptible to *P*. *falciparum* infection. Additionally, previous field studies from Burkina Faso [6] and Senegal [44] reported no difference in sporozoite rate between *An. gambiae* s.s. and *An. coluzzii* confirming the findings of the present study. Furthermore, a high proportion of homozygote mosquito to 1014F-genotype was found here in the sporozoite-infected mosquito sub-group compared to uninfected group, but there was no statistical difference suggesting that variation in resistance genotype proportion is not associated with the infection status. The current findings do not support the work from Alout et al. [23], who showed that *An. gambiae* mosquitoes with *kdr* alleles are more susceptible to *Plasmodium* than insecticide susceptible mosquitoes, and also contrast with the findings in Senegal, Burundi and Tanzania, which reported a significant correlation between the 1014F-genotype and the infection with *P. falciparum* [45–47]. This disagreement could be explained by the small sample size of this study which may limit the power to infer this relationship. The small sample size in this study also limited the comparison of the 1014F-genotype frequencies from mosquito collected as larvae versus those collected as adults. So, the results may have been different when increasing the samples size to those of Kabula et al. [47], who tested 526 specimens.

## Conclusion

The present study showed the diversity of malaria vectors species composition and significant variations in the *kdr* resistance genotype between vector species. However, associations were not found between 1014F-genotype and *P. falciparum* sporozoite infection status in Burkina Faso. Further work with a larger sample size of wild-caught mosquitoes is needed to establish the relationship between insecticide resistance in *An. gambiae* and ability to transmit *P. falciparum* in Burkina Faso, in the context of increasing malaria cases from year-to-year despite the scaling-up of the LLINs by the National Malaria Control Programme (NMCP).

## Additional files

**Additional file 1: Table S1.** The contrast in mean number of mosquito collected in each trap and the ratios between species, standard errors, z ratios and the p-values.

**Additional file 2: Table S2.** Species composition and relative frequency of the *An. gambiae* complex members by collection method and sites.

**Additional file 3: Table S3.** Ccomparison of the kdr resistant phenotype between species, p-values adjusted according to tukey methods.

## Abbreviations

CSP: circum-sporozoite protein
DNA: deoxyribonucleic acid
DDT: dichlorodiphenyl ethane
ELISA: enzyme linked immuno-sorbent assay
GLMMs: generalized linear mixed effect models
TNs: insecticide-treated nets
IRS: indoor residual spraying
Kdr: knockdown resistance
LLINs: long-lasting insecticide-treated nets
OR: odds ratio
PCR: polymerase chain reaction
PSC: pyrethroid spray catch
