# Supplementary material for: Anopheline species composition and the 1014F-genotype in different ecological settings of Burkina Faso in relation to malaria transmission
Source: Malar J. 2019 May 8;18:165. doi: 10.1186/s12936-019-2789-8 (PMC6507147; doi:10.1186/s12936-019-2789-8)
Supplement: Supplementary file 1 — Additional file 1: Table S1. The contrast in mean number of mosquito collected in each trap and the ratios between species, standard errors, z ratios and the p-values. [file 12936_2019_2789_MOESM1_ESM.docx]

**Table S1**: The contrast in mean number of mosquito collected in each trap and the ratios between species, standard errors, z ratios and the p-values.

| Species contrast | Methods | ratio | Std Error | z.ratio | p.value |
| --- | --- | --- | --- | --- | --- |
| *An. funestus sp / An. gambiae s.l.* | Exit Trap | 0.391 | 0.140 | -2.616 | 0.044 * |
| *An. funestus sp / Culex sp* | Exit Trap | 0.597 | 0.221 | -1.390 | 0.505 |
| *An. funestus sp / Other Anopheles sp* | Exit Trap | 0.834 | 0.339 | -0.447 | 0.970 |
| *An. gambiae s.l. / Culex sp* | Exit Trap | 1.527 | 0.236 | 2.739 | 0.031 * |
| *An. gambiae s.l. / Other Anopheles sp* | Exit Trap | 2.132 | 0.503 | 3.208 | 0.007 * |
| *Culex sp / Other Anopheles sp* | Exit Trap | 1.396 | 0.352 | 1.326 | 0.546 |
| *An. funestus sp / An. gambiae s.l.* | Pit-Shelter | 0.975 | 0.162 | -0.150 | 0.999 |
| *An. funestus sp / Culex sp* | Pit-Shelter | 1.354 | 0.224 | 1.829 | 0.260 |
| *An. funestus sp / Other Anopheles sp* | Pit-Shelter | 1.643 | 0.361 | 2.263 | 0.107 |
| *An. gambiae s.l. / Culex sp* | Pit-Shelter | 1.388 | 0.115 | 3.942 | < 0.001* |
| *An. gambiae s.l. / Other Anopheles sp* | Pit-Shelter | 1.685 | 0.281 | 3.126 | 0.010 * |
| *Culex sp / Other Anopheles sp* | Pit-Shelter | 1.214 | 0.202 | 1.165 | 0.649 |
| *An. funestus sp / An. gambiae s.l.* | PSC | 0.379 | 0.075 | -4.925 | < 0.001 * |
| *An. funestus sp / Culex sp* | PSC | 1.909 | 0.406 | 3.040 | 0.013 * |
| *An. funestus sp / Other Anopheles sp* | PSC | 1.427 | 0.378 | 1.343 | 0.536 |
| *An. gambiae s.l. / Culex sp* | PSC | 5.032 | 0.591 | 13.767 | < 0.001 * |
| *An. gambiae s.l. / Other Anopheles sp* | PSC | 3.761 | 0.756 | 6.589 | < 0.001 * |
| *Culex sp / Other Anopheles sp* | PSC | 0.747 | 0.159 | -1.365 | 0.522 |

* Show significance in difference
