# Supplementary material for: Anopheline species composition and the 1014F-genotype in different ecological settings of Burkina Faso in relation to malaria transmission
Source: Malar J. 2019 May 8;18:165. doi: 10.1186/s12936-019-2789-8 (PMC6507147; doi:10.1186/s12936-019-2789-8)
Supplement: Supplementary file 2 — Additional file 2: Table S2. Species composition and relative frequency of the An. gambiae complex members by collection method and sites. [file 12936_2019_2789_MOESM2_ESM.doc]

**Table S2:** Species composition and relative frequency of the *An. gambiae* complex members by collection method and sites

The number of specimens collected by each collection method is given and the relative proportion is in brackets. N is the total number of species collected according to locality. Proportions are compared between localities and between collection methods; the X2 value and P-values associated are given.
