# Supplementary material for: Anopheline species composition and the 1014F-genotype in different ecological settings of Burkina Faso in relation to malaria transmission
Source: Malar J. 2019 May 8;18:165. doi: 10.1186/s12936-019-2789-8 (PMC6507147; doi:10.1186/s12936-019-2789-8)
Supplement: Supplementary file 3 — Additional file 3: Table S3. Ccomparison of the kdr resistant phenotype between species, p-values adjusted according to tukey methods. [file 12936_2019_2789_MOESM3_ESM.docx]

**Table S3**: Ccomparison of the kdr resistant phenotype between species, p-values adjusted according to tukey methods.

| contrast | Odds.ratio | SE | z.ratio | p.value |
| --- | --- | --- | --- | --- |
| *An. arabiensis / An. coluzzii* | 0.5755 | 0.3077 | -1.0333 | 0.5558 |
| *An. arabiensis / An. gambiae* | 0.0166 | 0.0109 | -6.2679 | < 0.0001 |
| *An. coluzzii / An. gambiae* | 0.0289 | 0.0186 | -5.5070 | < 0.0001 |
